# The Role of Erythroid Differentiation Regulator 1 (ERDR1) in the Control of Proliferation and Photodynamic Therapy (PDT) Response

**DOI:** 10.3390/ijms21072603

**Published:** 2020-04-09

**Authors:** Sunyoung Park, Kyung Eun Kim, Hyun Jeong Park, Daeho Cho

**Affiliations:** 1 Kine Sciences, 525, Seolleung-ro, Gangnam-gu, Seoul 06149, Korea; sypark@kinesciences.com; 2 Department of Cosmetic Sciences, Sookmyung Women’s University, Chungpa-Dong 2-Ka, Yongsan-ku, Seoul 04310, Korea; kyungeun@sookmyung.ac.kr; 3 Department of Dermatology, Yeouido St. Mary’s Hospital, The Catholic University of Korea, Seoul 07345, Korea; 4 Institute of Convergence Science, Korea University, Anam-ro 145, Seongbuk-ku, Seoul 02481, Korea

**Keywords:** erythroid differentiation regulator 1 (ERDR1), photodynamic therapy (PDT), skin cancer, skin disease, apoptosis

## Abstract

Erythroid differentiation regulator 1 (ERDR1) was newly identified as a secreted protein that plays an essential role in maintaining cell growth homeostasis. ERDR1 enhances apoptosis at high cell densities, leading to the inhibition of cell survival. Exogenous ERDR1 treatment decreases cancer cell proliferation and tumor growth as a result of increased apoptosis via the regulation of apoptosis-related gene expression. Moreover, ERDR1 plays a pivotal role in skin diseases; ERDR1 expression in actinic keratosis (AK) is negatively correlated with the increase in apoptosis. Because of its high specificity and efficiency, photodynamic therapy (PDT) is a common therapy for patients with various skin diseases, including cancer. Many studies indicate that apoptosis is mainly induced by PDT treatment. As an apoptosis inducer, the recovery of the ERDR1 expression after PDT is correlated with good therapeutic outcomes. Here, we review recent findings that highlight the function of ERDR1 in the control of apoptosis. Thus, ERDR1 may have a role in the apoptosis regulation of target cells in the lesions, as the recovery of its expression after PDT is correlated with good therapeutic outcomes.

## 1. Introduction

Erythroid differentiation regulator 1 (ERDR1) was first reported as a hemoglobin synthesis-inducing factor in leukemia cells [1], and was subsequently identified as a secreted component that helps maintain cell survival during stressful conditions [2]. The cell density and ERDR1 concentration are dependent on the cell growth or cell death rate of BL-70 cells (Burkitt’s lymphoma cell line) [2]. Specifically, ERDR1 secretion significantly increases cell growth and survival at a low cell density or lower ERDR1 concentration. However, this factor suppresses cell growth and survival at high cell densities and at relatively higher ERDR1 concentrations in BL-70 cells. These data indicate that ERDR1 plays an essential role in maintaining cell growth homeostasis [2]. We reviewed and selected recent findings that highlight the function of ERDR1 as a growth control molecule that regulates apoptosis mediated by photodynamic therapy (PDT). Hence, we suggest that ERDR1 may be a potential biomarker for successful PDT through the regulation of apoptosis.

## 2. Photodynamic Therapy (PDT) for Disease Treatment

Photodynamic therapy (PDT) is a common procedural therapy for either malignant or benign dermatological lesions. PDT has advantages such as the selectivity of the photosensitizer towards target cells and minimal side effects, such as pain and burning [3]. Because of its specificity and selectivity, PDT has become a particularly attractive tool for cancer and as well as other skin disease treatment. The treatment results have been shown to achieve both good therapeutic effects and excellent cosmetic outcomes [4]. As normal tissue is barely damaged during PDT, it is a powerful tool for the treatment of various cancers, without the requirement of surgery. It can even be used for cancer patients in combination chemotherapy, so that the highly proliferative cells can be removed without surgery [5]. Currently, PDT is often used for superficial and premalignant skin conditions because of the limited light penetration and the increased depth of tumors. Previous studies have revealed that PDT is particularly effective for treating nonmelanoma skin cancers (NMSC), such as actinic keratosis (AK), squamous cell carcinoma (SCC), basal cell carcinoma (BCC), and Bowen’s disease, with high success rates and excellent cosmetic outcomes [6]. Moreover, PDT is well established for treating superficial BCC, even for relatively large lesions, such as nodular BCC, and its clinical outcomes are outstanding [4,7]. Until now, conventional PDT (cPDT) has been suggested and approved by the FDA and the EMA to cure mainly AK, Bowen’s disease, and nodular BCC [8].

Many studies indicate that the mitochondrial pathway and death receptor pathway are responsible for PDT-mediated apoptosis [3,9]. The application of a photosensitizer, a light absorbing molecule, onto the lesions, directly or systemically, is the beginning of PDT treatment. After penetration into the tissue, the photosensitizer accumulates in the target cells and subsequently induces cell death, following treatment with a light source with a specific wavelength that selectively affects the target cells as it is absorbed by the photosensitizer. When reactivated at a specific wavelength, the excited photosensitizer transforms its energy and stimulates photochemical reactions to produce radicals [3,9,10], which induces reactive oxygen species (ROS) production. The mechanisms underlying cell death after PDT are not entirely clear, although several studies have reported the involvement of apoptosis in target cells after treatment. One study revealed that mitochondrial ROS is critical for initiating mitochondrial inner membrane permeabilization and cytochrome c release, resulting in apoptotic cell death during PDT in A431 human epidermal carcinoma cells [11]. In this study, silicon phthalocyanine 4 (Pc 4), a second-generation photosensitizer, was efficiently localized to the mitochondrial membrane, and ROS generation was observed to increase dramatically within 5 min. Changes in the mitochondrial membrane potential, permeabilization, and cytochrome c release following PDT are responsible for apoptotic cell death, as confirmed in studies using A431 cells. Nakaseko et al. also showed histological changes in human skin tissue over time, on the induction of apoptosis after PDT [12]. In this study, apoptotic cells were observed via caspase-3 staining and terminal deoxynucleotidyl transferase-mediated deoxyuridine triphosphate-biotin nick end-labeling (TUNEL). Furthermore, in human nasopharyngeal carcinoma (NPC) cells, Fas-mediated apoptosis is induced by PDT [13]. CD95 signaling-dependent apoptotic cell death was observed in poorly differentiated (CNE2) and moderately differentiated (TW0-1) human NPC cells, followed by mitochondrial cytochrome c release and caspase-3 activation. Moreover, the susceptibility of target cells to apoptotic cell death by PDT is based on the balance between the pro- and anti-apoptotic members of the Bcl-2 family proteins, such that an increased Bax/Bcl-2 ratio is associated with increased apoptotic cell death [14]. More recently, in rat acute myeloid leukemia (LT12), apoptotic cell death events by PDT have been shown to be controlled over time through the regulation of apoptosis-related gene expression and caspase activation [15].

Altogether, PDT primarily destroys target cells by apoptosis as a result of ROS generation, caspase-3 activation, and a high Bax/Bcl-2 ratio. From the accumulated data, it can be inferred that PDT has a specific apoptosis-related mechanism that leads to good clinical outcomes.

## 3. Role of ERDR1 in PDT Response

A previous study suggested an essential role for ERDR1 in cell growth homeostasis, implying that ERDR1 regulates cell proliferation or cell death under certain conditions [2]. Based on studies that ERDR1 suppresses cell growth in high cell densities of Burkitt’s lymphoma (BL-70) cells [2], here, we describe the current evidence that ERDR1 may have a pro-apoptotic effect on uncontrolled proliferation, which may be the reason for successful PDT treatment.

### 3.1. ERDR1 Expression and Apoptosis Control

Dormer et al. reported that ERDR1 is a highly conserved protein that is expressed in normal human and mouse tissues [2]. Recent studies also show that the ERDR1 protein is highly expressed in normal skin tissues, and that its expression is significantly decreased in various skin diseases that are characterized by uncontrolled proliferation [16,17,18]. Specifically, the ERDR1 protein is highly expressed in keratinocytes and melanocytes, however, its expression is significantly decreased in the keratinocytes of patients with psoriasis and in the melanocytes of patients with malignant melanoma [16,18]. Additionally, ERDR1 protein levels are negatively correlated with skin malignancies, and its expression level in psoriasis and AK is often reduced, relative to normal controls, but is higher than that in skin tumors such as squamous cell carcinoma (SCC) or melanoma [17]. Overall, ERDR1 expression is responsible for the condition of the skin, i.e., normal levels of expression result in the maintenance of homeostasis and decreased levels lead to uncontrolled proliferation.

As the function of ERDR1 is the maintenance of homeostasis in stressful conditions, it has been reported that ERDR1 acts as a pro-apoptotic factor in human keratinocytes by mediating UVB-induced signal transduction [19]. In this study, ERDR1 gene knockdown using shRNA resulted in decreased UVB-induced apoptosis and caspase-3 activity, suggesting that UVB-induced apoptosis is dependent on ERDR1 expression in keratinocytes. More recently, it was reported to exert a pro-apoptotic effect on T cells via the activation of caspase-3 [20]. Microbiota suppresses the ERDR1 mRNA expression in CD3+CD4+ T cells, leading to the increased survival of T cells. The inhibition of the ERDR1 expression using ERDR1 shRNA in CD4+ T cells decreases the caspase-3 activation and Fas receptor expression, leading to the suppression of apoptosis [20]. These data suggest that ERDR1 plays a role in the pro-apoptotic factor by caspase-3 activation.

It has been reported that melanoma is extremely resistant to chemotherapy because of the low apoptotic rate in the cancer cells [21]. Recently, the ERDR1 protein was found to enhance apoptosis in melanoma cells by regulating Bcl-2 and Bax expression, providing evidence for its critical role in the suppression of melanoma progression [22]. Lee et al. confirmed that treatment with recombinant ERDR1 induces apoptosis via the downregulation of Bcl-2 and the upregulation of Bax in B16F10 cells in vitro [22]. Moreover, Jung et al. reported that ERDR1 overexpression leads to a reduction in cell migration, invasion, and proliferation in the murine melanoma cell line, B16F10 [16]. These investigators also reported that ERDR1-overexpressing melanoma cells have a decreased migratory ability, leading to the suppression of melanoma metastasis. Intraperitoneal injection of recombinant ERDR1 protein (100 μg/kg) enhanced apoptosis and significantly suppressed tumor growth in mouse melanoma B16F10 cell-implanted mice. In this study, ERDR1 overexpression surprisingly induces the downregulation of heat shock protein 90 (HSP90). As HSP90 serves as a marker of melanoma progression, several studies have suggested HSP90 inhibitors as therapeutic targets for treating cancers, including melanoma. HSP90 inhibitors increase apoptosis or autophagy in human melanoma cells [23,24]. These findings suggest that ERDR1 has a role in the control of apoptosis, either by regulating the expression of anti-apoptosis molecules (Bcl-2) and pro-apoptosis molecules (Bax), or by the downregulation of HSP90 in stressful conditions.

As seen in melanoma, ERDR1 also functions as an apoptosis regulator in several skin diseases. Kim et al. investigated the role of ERDR1 in psoriasis pathogenesis and suggested it as a therapeutic target [18,25]. Although ERDR1 is highly expressed in normal epidermal keratinocytes, its expression is significantly reduced in psoriatic patients, implying that ERDR1 downregulation plays an important role in the uncontrolled proliferation of keratinocytes in psoriasis [18]. It is confirmed that ERDR1 treatment effectively reduced psoriatic symptoms in imiquimod-induced psoriasis-like inflammation in mice [25]. More recently, it has been reported that the ERDR1 protein is negatively correlated with Bcl-2 and p53 expression, which leads to the abnormal proliferation of keratinocyte in the lesions of patients with AK [26]. In this report, Woo et al. investigated whether the low-level expression of ERDR1 in AK patients could be altered by treatment with ingenol mebutate, a common treatment for cutaneous neoplastic disorder such as AK. The results of the study show that the ERDR1 expression was significantly elevated following treatment with ingenol mebutate in AK lesional skin. Furthermore, the expression of the anti-apoptosis molecule Bcl-2 was also reduced in the lesional skin, indicating that the ERDR1 expression recovery induced by this effective treatment of AK is correlated with the enhancement of apoptosis through the downregulation of Bcl-2.

Collectively, these findings suggest that ERDR1 controls cell fate by inducing apoptosis under certain conditions in the skin characterized by uncontrolled proliferation, including melanoma. Helping the recovery of ERDR1 levels, specifically in conditions with decreased expression, such as psoriasis, AK, SCC, or melanoma, may be a promising way to overcome these abnormal conditions via the induction of apoptosis.

### 3.2. ERDR1 and Photodynamic Therapy

PDT is more advantageous than chemotherapy or radiotherapy because of its minimal side effects during treatment. Nevertheless, PDT is a widely used therapy to treat various skin diseases and cancers; however, poor results or limitations are still present. One reason is that skin cancers contain senescent cells [27]. Senescent cells accumulate with age and are resistance to apoptosis, resulting in therapy-resistance. Grigalavicius et al. investigated the influence of PDT with 5-aminolevulinic acid (ALA) on senescent cells. The results have indicated that PDT–ALA effectively inactivates senescent WM115 human primary melanoma cells. They have suggested the use of PDT-ALA for killing senescent cells and increasing the efficacy of anticancer therapy. Another reason is that several tumor survival factors are sometimes activated after PDT [28]. Broekgaarden et al. reviewed several tumor survival pathways activated by PDT. The authors explained that ROS produced by PDT also triggers a stress response that helps cancer cell survival. For example, the transcription factor activator protein 1 (AP-1), nuclear factor E2-related factor 2 (NRF2), hypoxia-inducible factor 1 (HIF-1), and nuclear factor κB (NF-κB) are the main triggers of PDT-surviving cells. These tumor survival pathways cause the antioxidant stress response, modifying and altering the gene expression to create a tumor-friendly microenvironment.

Because PDT is still most often used for superficial and premalignant skin conditions because of the limited light penetration and the increased depth of the tumor, studies on how to overcome this must be conducted. First, pain associated with PDT treatment should be considered. Daylight PDT (dPDT) other than cPDT results in less pain and is recommended [6]. The differences between cPDT and dPDT are the light sources and treatment-related pain. Usually, red or blue wavelengths are required for dPDT in daylight. As the time required to penetrate the skin is much longer during the cPDT process, the applied photosensitizers are mainly located in deep tissue where cutaneous nerves are found, which causes moderate to severe pain [29]. It seems that dPDT results in patient satisfaction because of the almost zero pain as well as excellent cosmetic results upon treatment [30,31]. Next, Ferrario et al. suggested that targeting survivin, a Hsp-90 client protein, possibly improves PDT responses [32]. They found that survivin is increased by PDT. The combination of PDT with the geldanamycin derivative, 17-AAG, which causes proteasome degradation and competes with Hsp-90 client proteins to bind to Hsp-90, dramatically reduces survivin expression in malignant human BT-474 breast cancer cells. As a result, the Bcl-2 expression is downregulated and the PDT responses are enhanced. Another report by Ferrario et al. revealed that PDT efficacy can be improved by targeting HSP90 in BA mammary carcinoma cells [33]. When 17-AAG was combined with PDT, survivin expression was dramatically reduced along with Akt pathway inactivation. Pro-survival factors such as HIF-1α, VEGF, MMP9, and MMP2 were also reduced by 17-AAG combined with PDT. They confirmed these results using a BA tumor-bearing mouse model, and, as expected, 17-AAG enhanced the PDT treatment of BA tumors in comparison with PDT monotherapy. They suggested that a combined modality may be beneficial for PDT. More recently, it has been elucidated that mitochondria targeting sensitizers are likely to increase PDT efficacy [34]. Thomas et al. introduced IR-PU, a heat shock protein inhibitor-mitochondria targeting an indocyanine dye conjugate for selective accumulation on target cells. IR-PU is designed to bind HSP90 family proteins that are overexpressed in cancer cells, sometimes because preferential accumulation in target cells is not guaranteed [35]. IR-PU efficiently accumulated only in cancer cell lines (HeLa (human cervical cancer cell line) and HCI-H460 (human lung cancer cell line)) and not in normal hepatocytes. It strongly inhibits Hsp90 client proteins, Chk1, and induces apoptosis through caspase 3 activation and PARP1 cleavage [34]. They suggested that direct mitochondrial targeting and accumulation could affect mitochondrial membrane depolarization and improve PDT efficacy.

**Figure 1:**
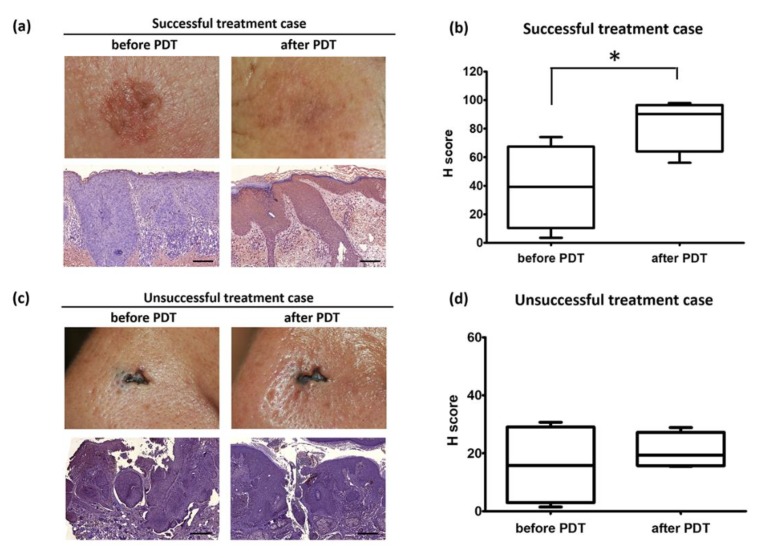
Comparison of erythroid differentiation regulator 1 (ERDR1) expression following photodynamic therapy (PDT) in successful and unsuccessful treatments. (**a**) Images of lesions of actinic keratosis (AK) patients before and after PDT (top panels) and immunohistochemical staining of ERDR1 in skin tissue samples (lower panels). Scale bar = 10 µm. (**b**) ERDR1 expression of four patients with successful PDT treatment quantified by digital image analysis, and an H-score (Histoscore) was calculated. To quantify the intensity of the ERDR1 expression, digital images of ERDR1-immunostained tissues were obtained using a panoramic scan slide scanner (3D HISTECH, Budapest, Hungary), and analyzed using the HistoQuant software (3D HISTECH) with the DensitoQuant algorithm (3D HISTECH). The DensitoQuant algorithm shows five different color images assigned according to staining intensity (red, orange, yellow, blue, and white, indicating strongly positive pixels, moderately positive pixels, weakly positive pixels, negative pixels, and the hematoxylin background, respectively). The algorithm automatically calculates the ratio of positive to total pixels by the formula [1 × (% of weakly positive pixels) + 2 × (% of moderately positive pixels) + 3 × (% of strongly positive pixels)] to provide an H-score. An unpaired t-test was performed to analyze the intensity of the ERDR1 expression between the two groups before and after PDT. The results represent the mean ± standard error of the mean (SEM) and H-score from four different donors. *denotes statistically significant (*p* < 0.05) changes from before PDT. (**c**) Images of lesions of BCC patients before and after PDT (top panels), and immunohistochemical staining of ERDR1 in skin tissue (bottom panels). Scale bar = 10 µm. (**d**) The ERDR1 expression of four patients with unsuccessful PDT treatment was quantified. Data for H-scores are shown as mean ± SEM.

Given that PDT induces target cell death by apoptosis, we hypothesized that ERDR1 could promote apoptosis in response to PDT. Like targeting survivin, ERDR1 can affect PDT-mediated apoptosis to improve PDT response as a result of successful treatment, because ERDR1 functions as an apoptosis inducer, as mentioned before. As shown in Figure 1, the expression of ERDR1 in the skin tissues from AK patients was dramatically increased after PDT compared to the levels before PDT, and the tissue exhibited a good cosmetic result following treatment (Figure 1a). The average intensity of the ERDR1 expression was significantly upregulated after PDT compared with the levels before PDT, as analyzed by an unpaired t-test (*p* = 0.03; Figure 1b). This change implies that the ERDR1 expression after PDT somehow recovered to levels similar to those that are present normally, because the ERDR1 expression is decreased in various skin diseases that are characterized by uncontrolled proliferation, such as AK, psoriasis, and cancer [16,17,18]. ERDR1 protein is highly expressed in keratinocytes and melanocytes, however, its expression is significantly decreased in keratinocytes from patients with psoriasis and in melanocytes from patients with malignant melanoma [16,18]. Additionally, ERDR1 protein levels are negatively correlated with skin malignancies, and its expression level in psoriasis and AK is often reduced relative to normal controls, but is higher than that in skin tumors, such as SCC or melanoma [17]. Therefore, it seems that ERDR1 expression is closely linked to the condition of the skin, wherein normal levels of expression result in maintenance of skin homeostasis and decreased levels lead to uncontrolled proliferation. Thus, it is thought that ERDR1 induces PDT-mediated apoptosis and is related to good clinical results, as seen in successful treatment cases (Figure 1a,b). However, the recovery of ERDR1 levels after PDT was not detected in a patient with other non-melanoma skin cancers, such as Bowen’s disease and BCC, but the PDT outcome was also not successful (Figure 1c,d). In this unsuccessful treatment case, the lack of recovery of ERDR1 expression in the lesion and the insufficient apoptosis of target cells may explain the failure of PDT to control the neoplasm.

**Figure 2:**
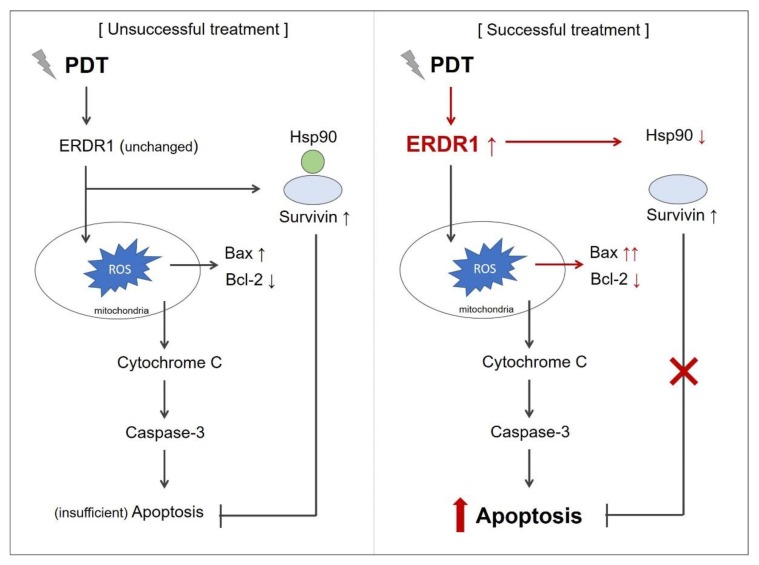
Schematic picture of photodynamic therapy (PDT) response mediated by ERDR1. In the case of unsuccessful treatment, the ERDR1 expression level after PDT was not changed after PDT. Survivin increased by PDT is one reason to show insufficient apoptosis. As shown in the successful treatment case, the increased expression of ERDR1 after PDT may reduce the Hsp90 expression, resulting in the inhibition of surviving/Hsp90 complex formation. In addition, the recovery of ERDR1 may increase the Bax/Bcl-2 ratio to activate caspase-3. Altogether, ERDR1 is suggested as a key factor of PDT-mediated apoptosis to improve PDT response.

Eventually increasing the PDT response is a key factor in successful treatment, and a strategy to stimulate pro-apoptotic molecules is one of the ways to improve PDT effectiveness and overcome the limitations. Here, as per our knowledge, increasing the ERDR1 expression is suggested to increase PDT-mediated apoptosis, which has been reported for the first time. Furthermore, the increased expression of ERDR1 after PDT may regulate the Bcl-2/Bax ratio and/or Hsp-90/survivin interaction, resulting in the activation of caspase-3 (Figure 2). Further studies will be required to elucidate the specific mechanism of the recovery of ERDR1 through PDT and the involvement of ERDR1 in PDT-mediated apoptosis.

## 4. Materials and Methods

### 4.1. Skin Biopsy Specimens and Immunohistochemistry

Skin biopsy specimens were obtained from AK patients (*n* = 4), patients with Bowen’s disease (*n* = 2), and BCC patients (*n* = 2) who were recruited from Yeouido St. Mary’s Hospital, The Catholic University of Korea. All of the studies using human subjects were approved by the ethics committee of the Catholic University of Korea. All of the skin tissues were fixed with 4% paraformaldehyde and embedded in paraffin, and paraffin sections were produced. To detect the ERDR1 expression, the tissue sections were blocked with a blocking solution containing 5% goat serum, and were incubated for 2 h at room temperature with a polyclonal rabbit anti-human ERDR1 antibody (1:1000 dilution). After washing the sections, HRP-conjugated goat anti-rabbit IgG was added as a secondary antibody. The substrate used to detect the ERDR1 expression was 3,3′-Diaminobenzidine (DAB; Invitrogen Life Technologies, Carlsbad, CA, USA).

### 4.2. Digital Images of Immunostained Tissues

To quantify the intensity of the ERDR1 expression, digital images of the ERDR1-immunostained tissues were obtained using a panoramic scan slide scanner (3D HISTECH, Budapest, Hungary) and analyzed using the HistoQuant software (3D HISTECH) with the DensitoQuant algorithm (3D HISTECH). The DensitoQuant algorithm shows five different color images assigned according to the staining intensity (red, orange, yellow, blue, and white, indicating strongly positive, moderately positive, weakly positive, negative, and hematoxylin backgrounds, respectively). The algorithm automatically calculates the ratio of positive to total pixels, using the formula [1 × (% of weakly positive pixels) + 2 × (% of moderately positive pixels) + 3 × (% of strongly positive pixels)] to provide an H-score.

### 4.3. Statistical Analysis

All of the values were analyzed with an unpaired Student’s t-test, and the statistical analysis was performed using GraphPad Prism 8 (GraphPad Software, San Diego, CA, USA). *P* values < 0.05 were considered statistically significant in a two-tailed t-test.

## 5. Conclusions

PDT is a procedural therapy for patients with skin diseases, because of its high specificity and efficiency. The application of a photosensitizer accumulates in the target lesion, and a light source with a specific wavelength stimulates ROS production, which in turn causes target cell death responsible for PDT-mediated apoptosis. Recent studies provide accumulating evidence that ERDR1 functions as an apoptosis regulator to control homeostasis. ERDR1 enhances apoptosis at high cell densities of the Burkitt’s lymphoma cell line (BL-70), leading to the inhibition of cell survival. Additionally, exogenous ERDR1 treatment decreases cancer cell proliferation and tumor growth in melanoma as a result of increased apoptosis via the regulation of apoptosis-related molecules—Bcl-2 and Bax. In addition to melanoma, ERDR1 plays a pivotal role in some skin diseases, such as psoriasis and AK, which are characterized by uncontrolled cell proliferation. Specifically, the ERDR1 expression in AK is correlated with the enhancement of apoptosis through the downregulation of Bcl-2. Furthermore, the recovery of the ERDR1 expression after PDT is correlated with good therapeutic outcomes, and, at least in part, recovery is related to the apoptosis of target cells in the lesions, suggesting that this factor may have potential as a biomarker for successful PDT and be involved in apoptotic cell death in these situations.
